# Micro—Organisms in Clothing

**Published:** 1891-03

**Authors:** 


### Micro-Organisms in the Clothing.

Dr. Hobein, *Zeitschr. f. Hygiene*, has made a series of experiments to determine what class of goods used for under-clothing most readily take up organisms when worn over the skin, and to discover the conditions which increase or diminish their receptibility. He found that flannel goods contained more organisms than other fabrics on account of their thickness and rough surface; next as regards the number of contained microbes, were thin woolen goods, which are thinner than flannels, but more loosely woven and have a rougher surface. The smallest number of microbes was found in linen and cotton goods, which are closely woven and have a smooth surface.

The investigations further serve to show that under ordinary circumstances no increase of the organisms take place in the clothing. A marked development of the microbes on the skin or in the clothing only occurs when, owing to diminished evaporation, the skin and clothing are left moist for a long time.—*Internat. Pharmac. General-Anz.*, No. 32, 1890.

